# Chlorine Dioxide: Friend or Foe for Cell Biomolecules? A Chemical Approach

**DOI:** 10.3390/ijms232415660

**Published:** 2022-12-10

**Authors:** Celia María Curieses Andrés, José Manuel Pérez de la Lastra, Celia Andrés Juan, Francisco J. Plou, Eduardo Pérez-Lebeña

**Affiliations:** 1 Hospital Clínico Universitario, Avenida de Ramón y Cajal, 3, 47003 Valladolid, Spain; 2 Institute of Natural Products and Agrobiology, CSIC-Spanish Research Council, Avda. Astrofísico Fco. Sánchez, 3, 38206 La Laguna, Spain; 3 Cinquima Institute and Department of Organic Chemistry, Faculty of Sciences, Valladolid University, Paseo de Belén, 7, 47011 Valladolid, Spain; 4 Institute of Catalysis and Petrochemistry, CSIC-Spanish Research Council, 28049 Madrid, Spain; 5 Sistemas de Biotecnología y Recursos Naturales, 47625 Valladolid, Spain

**Keywords:** chlorine dioxide, toxicity, “miracle mineral solution”, human consumption

## Abstract

This review examines the role of chlorine dioxide (ClO_2_) on inorganic compounds and cell biomolecules. As a disinfectant also present in drinking water, ClO_2_ helps to destroy bacteria, viruses, and some parasites. The Environmental Protection Agency EPA regulates the maximum concentration of chlorine dioxide in drinking water to be no more than 0.8 ppm. In any case, human consumption must be strictly regulated since, given its highly reactive nature, it can react with and oxidize many of the inorganic compounds found in natural waters. Simultaneously, chlorine dioxide reacts with natural organic matter in water, including humic and fulvic acids, forming oxidized organic compounds such as aldehydes and carboxylic acids, and rapidly oxidizes phenolic compounds, amines, amino acids, peptides, and proteins, as well as the nicotinamide adenine dinucleotide NADH, responsible for electron and proton exchange and energy production in all cells. The influence of ClO_2_ on biomolecules is derived from its interference with redox processes, modifying the electrochemical balances in mitochondrial and cell membranes. This discourages its use on an individual basis and without specialized monitoring by health professionals.

## 1. Introduction

Regarding chlorine dioxide (ClO_2_), most of the reviews in the technical literature have been carried out from a biological or medical point of view, and others have analyzed its efficacy and safety as a disinfectant or in drinking water treatment. In this review, we focus on the chemical study of the interaction of ClO_2_ with organic molecules or inorganic cations present in cells, giving an overview of its reactivity, its potential toxicity for biological molecules and hazardousness if not used correctly, and cases for which it has been approved.

Chlorine dioxide was discovered in 1811 by Sir Humphry Davy and since the mid-20th century, it has been widely used in the paper industry as a bleach and for the treatment of drinking water. More recent developments extend its application to food processing, disinfection of premises and vehicles, mold eradication, air disinfection and odor control, swimming pool treatment, wound cleaning, and dental applications [1].

Chlorine dioxide (ClO_2_) is classified by the World Health Organization (WHO) as a safe and effective fourth generation, broad-spectrum, class A1 disinfectant [2,3]. It is used to purify drinking water without creating harmful concentrations of disinfection by-products [4]. The properties of ClO_2_ result from one-electron transfer reactions, so it is considered a strong oxidizing agent [5] and, unlike chlorine, does not tend to react with organic materials to form chlorinated species or with ammonia to form chloramine. Chlorine dioxide is an important biocide and bleach and is used as an alternative to chlorine in the purification and disinfection of drinking water [6]. ClO_2_ is used in 8.1% of drinking water treatment plants in the USA and 32.8% of those in China [7,8], and in some European countries [9], it is used in paper bleaching, sterilization of medical devices, and disinfection of foodstuffs [10]. According to the EPA, ClO_2_ is used “in public water-treatment facilities, to make water safe for drinking.” When chlorine dioxide is added to drinking water, it helps to destroy bacteria, viruses, and some types of parasites that can make people sick, such as *Cryptosporidium parvum* and *Giardia lamblia*.

**Figure 1:**
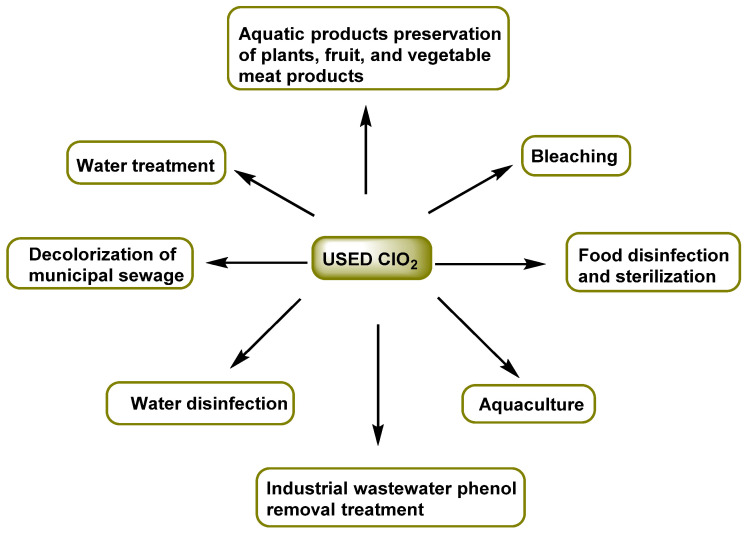
Applications of chlorine dioxide.

Its main advantage over chlorine is that it reduces the formation of harmful organochlorine compounds [11,12,13,14,15,16,17]. ClO_2_ is beneficial in minimizing the formation of trihalomethanes; however, ClO_2_ is converted to ClO_2_^−^ and ClO_3_^−^, which can cause hemolytic anemia and other health effects. The Environmental Protection Agency (EPA) has set the maximum concentration in drinking water at 0.8 milligrams per liter (mg/L) for chlorine dioxide and 1.0 mg/L for chlorite ions [18]. Some of its industrial applications are listed in Figure 1.

Chlorine dioxide is a compound that differs from elemental chlorine, both in its chemical structure and in its behavior [19]. An important characteristic is its high solubility in water, especially in cold water. Chlorine dioxide is about 10 times more soluble in water than chlorine.

## 2. Physicochemical Properties of ClO_2_

ClO_2_ is a yellowish-green gas and has a pungent odor, like Cl_2_, with a boiling point of 11 °C, a melting point of −59 °C, a density of 1.64 g mL^−1^ (liquid) at 0 °C, a water solubility of 3 g L^−1^ at 25 °C, and a pKa = 3. ClO_2_ is very soluble in water and does not hydrolyze, remaining in solution as a dissolved gas [20]. Solutions of ClO_2_ in water are stable when protected from light and kept at room temperature or below, well-sealed, and slightly acidified (pH = 6). The ultraviolet absorption spectrum of ClO_2_ solutions is broadband with a peak at 359 nm and a molar extinction coefficient of 1250 M^−1^ cm^−1^. ClO_2_ has a relatively short half-life and is highly volatile and explosive at concentrations > 10% in air [21]. Chlorine dioxide may not be compressed, stored, or transported under pressure and must be manufactured at the place of consumption.

**Figure 2:**
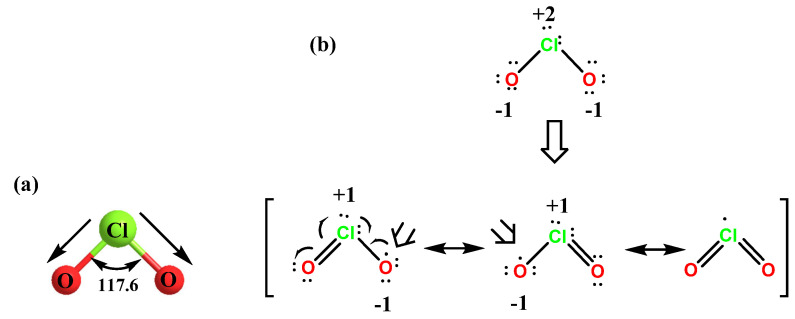
(**a**) The molecular structure of ClO_2_. (**b**) The Lewis structure of ClO_2_.

ClO_2_ is a neutral monomeric free radical with a dipole moment of 1.792 Debye [22]. From the microwave spectra of gas-phase chlorine dioxide, the chlorine-oxygen distance is found to be approximately 0.147 nm and electron diffraction indicates 0.149 nm. This chlorine-oxygen distance is approximately that of an average chlorine-oxygen double bond. Studies on the geometry of ClO_2_ established that the bond distance between the Cl atom and the O atom is smaller compared to the bond in chlorine monoxide (ClO). These results explain and justify the representation of the double bond between these two atoms, as well as showing that resonance structures satisfactorily explain the unpaired electron of the chlorine atom. ClO_2_ has a molecular geometry with an oxygen-chlorine-oxygen bond angle of 117.6°, as shown in Figure 2. In its ground state, although the unpaired electron is shared between the two oxygen atoms and the chlorine atom, most of the electron density resides mainly on either oxygen atom.

It has an odd number of valence electrons (it is a paramagnetic radical), and its electronic structure has long puzzled chemists, because none of the possible Lewis structures are satisfactory. In 1933, Brockway proposed a structure involving a three-electron bond [23]; Linus Pauling later developed this idea and proposed two possible resonant structures involving a double bond on the one hand and a single bond with a three-electron bond on the other [24].

The electronegativity of the two oxygen atoms is large enough to eliminate the electron density of the chlorine atom and gives chlorine a partial positive charge, Figure 2.

## 3. Generation of Chlorine Dioxide

Chlorine dioxide is a widely used disinfectant as an alternative to chlorine, due to its effectiveness in pathogen inactivation and low production of halogenated organic by-products of disinfection. However, during the generation of ClO_2_, chlorine is inevitably introduced into the ClO_2_ solution obtained as an impurity. The presence of chlorine in chlorine dioxide may affect the formation and toxicity of disinfection by-products as well as the disinfection efficiency.

**Figure 3:**
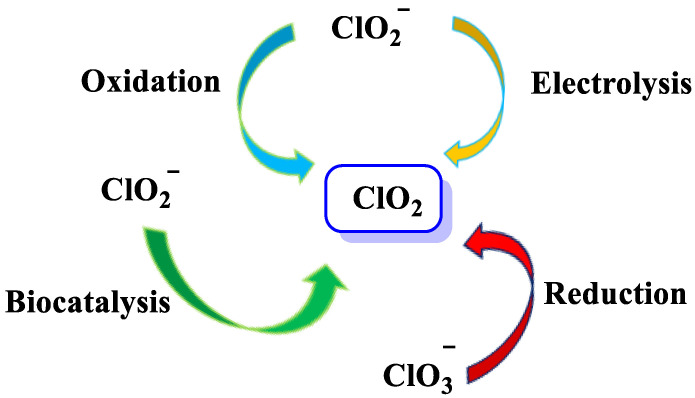
ClO_2_ preparation methods.

There are different methods for the preparation of chlorine dioxide [16], depending on the amount required, the number of by-products that can be tolerated, and whether the gas is required in solution or in gaseous form, Figure 3.

### 3.1. From Chlorite Ions

**Figure 4:**
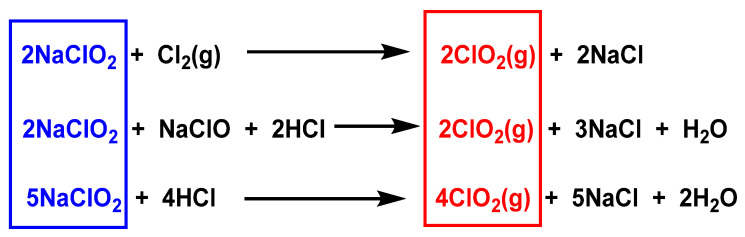
Preparation of chlorine dioxide from sodium chlorite.

ClO_2_ is generated from chlorite ions using chemicals, electrochemicals, and biocatalysts, and from the reaction of chlorite with chlorine gas Cl_2_ or hydrochloric acid (HCl) [25,26,27], as shown in Figure 4.

The methods described in Figure 4 have major disadvantages due to the production of high amounts of chlorides, which can be avoided by replacing hydrochloric with sulfuric acid, although in such cases, the processes become less efficient. These methods involve concentrated acids and/or externally added oxidants such as Cl_2_, OCl^−^, and H_2_O_2_.

Another way to generate ClO_2_ from chlorite by one-electron transfer is by electrochemical means [28], but this procedure requires a considerable input of electrical energy. An electrochemical method using mixed metal oxide MMO electrodes in the presence of chlorite and boron-doped diamond BDD anodes to promote the evolution of chlorine species was also studied [29,30]. Another possibility is to start from an undoped solution of sodium chlorite and a mixture of sodium chloride in an undivided electrochemical cell with a constant current, Ti/IrO2 anode, and Ti/Pt cathode [31,32].

To oxidize chlorite to ClO_2_, catalysts are based on manganese or iron porphyrin complexes. In these systems, chlorite dismutation is initiated through the oxidation of Mn(II or III) or Fe(III) by chlorite ions to produce hypochlorite ions and high-valent Mn and Fe(IV or V). Both oxidation states, IV and V, oxidize chlorite directly to ClO_2_, although complete conversion of chlorite to ClO_2_ was achieved in water using water-soluble Fe or Mn porphyrins. The synthesis of these ligands and catalysts is very expensive. A catalytic process has also been developed using a manganese porphyrin catalyst, tetra-kis-5,10,15,20-(N,N-dimethylimidazolium) porphyrinatomanganese(III), which is soluble in water and catalyzes the formation of chlorine dioxide from chlorite at room temperature and pH = 5 [33,34,35,36].

### 3.2. From Sodium Chlorate

Currently, the most widely used method to produce chlorine dioxide is the reduction method, by reacting sodium chlorate in a concentrated acid solution with reducing agents such as sulfur dioxide, methanol, oxalic acid, hydrogen peroxide, hydrochloric acid, or sodium chloride. With hydrochloric acid, the chlorine content is high, the purity of the chlorine dioxide is low, and the contamination is severe [37,38].

The sulfur dioxide method has the disadvantage of SO_2_ [38], with side reactions and low efficiency, which limits its application. The methanol process is currently the most widely used method for chlorine dioxide production in new-build plants worldwide [37]. The chlorine dioxide obtained is of high purity, but this method requires high acidity, and the reactor needs materials with excellent corrosion resistance.

**Figure 5:**
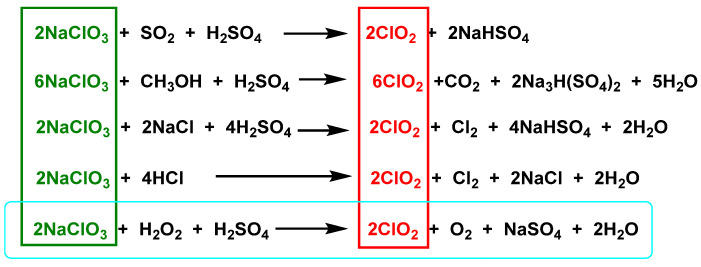
Preparation of chlorine dioxide from sodium chlorate.

In the chlorate reduction method, hydrogen peroxide advantageously replaces the other reagents, the process is more environmentally friendly, and the main by-product formed is oxygen, Figure 5.

**Figure 6:**
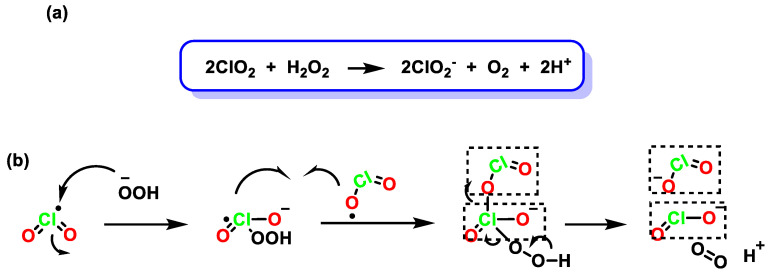
(**a**) Reduction of chlorine dioxide with hydrogen peroxide. (**b**) Reduction mechanism of chlorine dioxide with hydrogen peroxide.

The reaction between commercial solutions of chlorate and H_2_O_2_ results in the formation of ClO_2_ [38]. The reaction is very reproducible and stoichiometric. It is very important that the reaction mixture is not depleted of chlorate to avoid further reduction of ClO_2_. Once ClO_2_ is formed, the reduction of the chlorinated species continues, leading to the formation of other species, such as chlorite, Figure 6.

When large quantities of chlorine dioxide are needed, sodium chlorate is used as a raw material, and this method has traditionally been used in the pulp and paper industries. The conditions for producing ClO_2_ from sodium chlorite can be better controlled than those for sodium chlorate, but chlorite is more expensive and unstable, and therefore, from an industrial point of view, sodium chlorate is a more suitable feedstock [39].

## 4. Decomposition of ClO_2_

### 4.1. Disproportionation of Chlorine Dioxide with OH^−^

**Figure 7:**
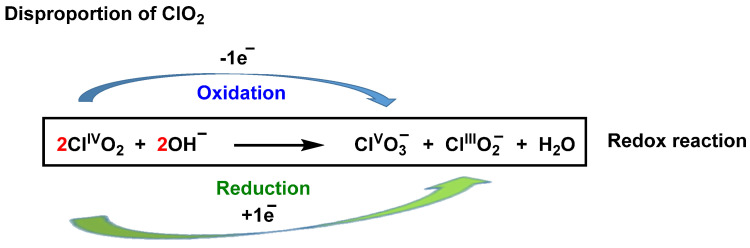
ClO_2_ as an oxidizing and reducing agent.

In solution at neutral pH, in the absence of light, and at room temperature or below, chlorine dioxide is fairly stable [40], but its decomposition is accelerated in alkaline solution to give ClO_2_^−^ and ClO_3_^−^ [41], Figure 7.

**Figure 8:**
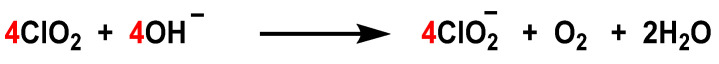
Molar stoichiometry from ClO_2_ to ClO_2_^−^.

Ion chromatography shows that ClO_2_^−^ and ClO_3_^−^ are the only chlorine products formed from the decomposition of ClO_2_ in a basic solution. However, the ratio of ClO_2_^−^ to ClO_3_^−^ is not 1:1 as required for the disproportionation reaction. According to several authors, the percentage of ClO_2_^−^ is higher than that of ClO_3_^−^ as the ClO_2_ concentration decreases. At micromolar levels of ClO_2_ the yield of ClO_2_^−^ is higher than that of ClO_3_^−^. The following additional reaction could explain the change of molar stoichiometry from ClO_2_^−^ to ClO_3_^−^ [42], Figure 8.

**Figure 9:**
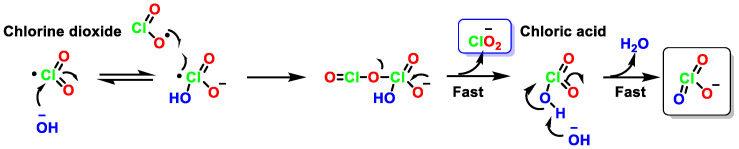
Mechanism of formation of equimolar amounts of ClO_2_^−^ and ClO_3_^−^ from ClO_2_ in basic medium.

**Figure 10:**
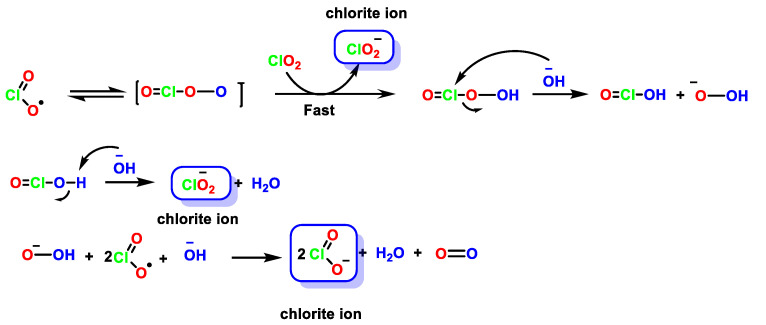
Mechanism of formation of ClO_2_^−^ from ClO_2_ in basic medium.

**Figure 11:**
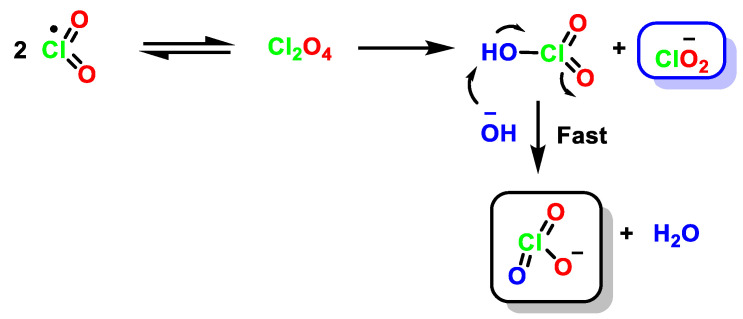
Mechanism of the formation of equimolar amounts of ClO_2_^−^ and ClO_3_^−^ from Cl_2_O_4_ in basic medium.

The three possible mechanisms (Figure 9, Figure 10 and Figure 11) can explain the stoichiometry of the decomposition of ClO_2_ in alkaline solution, via assisted electron transfer [41].

In the mechanism of ClO_3_^−^ formation from ClO_2_ in basic media, the reaction of ClO_2_ with OH^−^ generates species where OH^−^ binds to the Cl atom of ClO_2_ to form the intermediate (HOCl(O)O)^−^. The formation of ClO_3_^−^ occurred from the reaction between HOClO_2_ and OH^−^. This pathway shows first order kinetics with respect to the concentrations of ClO_2_ and OH^−^, Figure 9.

For the formation of ClO_2_^−^ in basic media, it is proposed that OH- forms an adduct with one of the oxygen atoms of ClO_2_ to give OClOOH^−^, and OCl is weakly bound to OOH. This adduct can undergo rapid electron transfer with a second ClO_2_ to give ClO_2_^−^ and OClOOH. The latter species reacts favorably with OH^−^ to generate HOClO and HOO^−^. The reaction between HOO^−^ and ClO_2_ gives ClO_2_^−^ and O_2_ [43], Figure 10.

A third possibility involves the formation of an intermediate dimer [44], Cl_2_O_4_, which reacts with OH^−^ (an electron transfer step). This pathway is important at high ClO_2_ concentrations, Figure 11.

The distribution of chlorine dioxide decomposition products in a basic solution changes as the ClO_2_ concentration decreases. While disproportionation reactions giving equal amounts of ClO_2_^−^ and ClO_3_^−^ dominate the stoichiometry at millimolar or higher ClO_2_ levels, the ratio of ClO_2_^−^ to ClO_3_^−^ formed increases significantly at micromolar levels of ClO_2_ [42].

**Figure 12:**
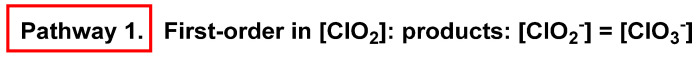
First order disproportionation reaction in [ClO_2_].

The kinetic evidence shows three concurrent pathways that show a first order dependence on [OH^−^] but have a variable order on [ClO_2_]. Pathway 1 is a disproportionation reaction that is first order in [ClO_2_], Figure 12.

**Figure 13:**
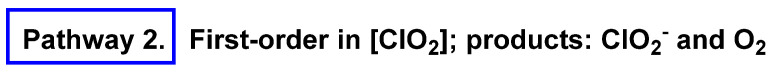
Pathway 2 is also first order in [ClO_2_] but forms ClO_2_^−^ as the only chlorine-containing product.

Pathway 2, a previously unknown reaction, is also first order in [ClO_2_] but forms ClO_2_^−^ as the only chlorine-containing product. Pathway 2 is attributed to the attack of OH^−^ on an oxygen atom of ClO_2_ leading to intermediate peroxide intermediates and producing ClO_2_^−^ and O_2_ as products. This pathway is important at low levels of ClO_2_, Figure 13.

**Figure 14:**
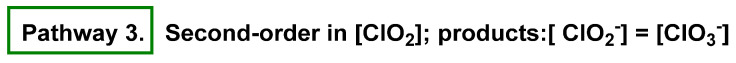
Pathway 3 is second order in [ClO_2_] and generates equal amounts of ClO_2_^−^ and ClO_3_^−^.

Pathway 3 is second order in [ClO_2_] and generates equal amounts of ClO_2_^−^ and ClO_3_^−^. A Cl_2_O_4_ intermediate is proposed for this pathway. At high ClO_2_ concentrations, pathway 3 brings the overall yield of ClO_3_^−^ close to the overall yield of ClO_2_^−^, Figure 14.

### 4.2. Disproportionation of Chlorine Dioxide with Nucleophile

**Figure 15:**
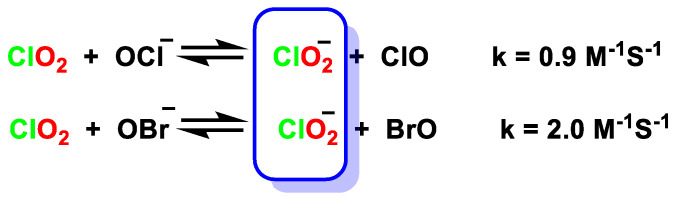
Effect of the OX^−^ hypohalite ion on the disproportionation of ClO_2_ in basic solution.

The effect of OX^−^ hypohalite ion catalysis on the disproportionation of chlorine dioxide in basic solution to give ClO_2_^−^ and ClO_3_^−^ has been studied. In the first step of hypohalite catalysis (the reaction between ClO_2_ and OX^−^ involved a transfer of electrons to form ClO_2_^−^ and OX), this step is reversible [45,46,47], Figure 15.

**Figure 16:**
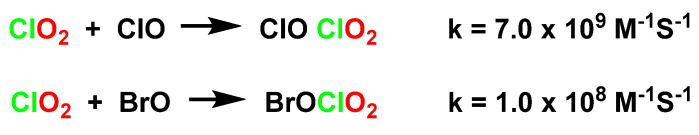
Formation of XOClO_2_.

In the second step, the reactions between ClO_2_ and XO form XOClO_2_, Figure 16.

**Figure 17:**
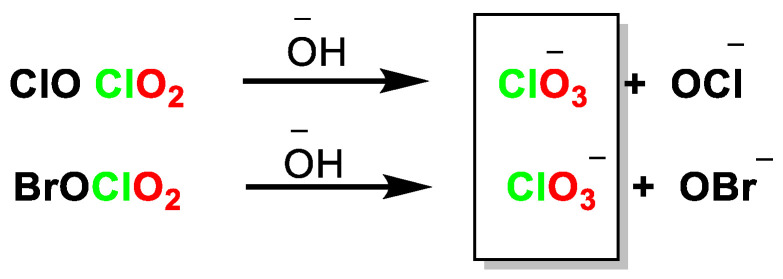
Hydrolysis of XOClO_2_ in basic medium to produce ClO_3_^−^ and OX^−^.

In basic medium, hydrolysis of XOClO_2_ produces ClO_3_^−^ and OX^−^, Figure 17.

### 4.3. Photodissociation of ClO_2_

**Figure 18:**
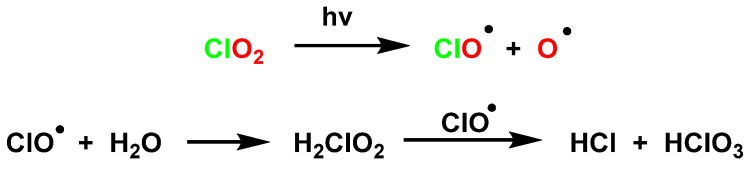
The homolysis of ClO_2_ in neutral aqueous solution.

The reactivity of ClO_2_ is modified by exposure to UV radiation in a process known as UV/ClO_2_. ClO_2_ undergoes photodissociation leading to the formation of the primary radical oxygen (O^•^), chlorine (Cl^•^), and chlorine oxide (ClO^•^) by homolytic fission of the chlorine-oxygen bond to form ClO^•^ and O^•^ [48,49,50]. Illumination of a neutral aqueous ClO_2_ solution gives a mixture of chloric acid and hydrochloric acid, Figure 18.

**Figure 19:**
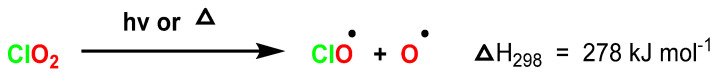
Homolysis of ClO_2_ by thermal route or with irradiation.

The photochemical and thermal decomposition of ClO_2_ takes place by homolytic fission of the chlorine-oxygen bond, Figure 19.

**Figure 20:**
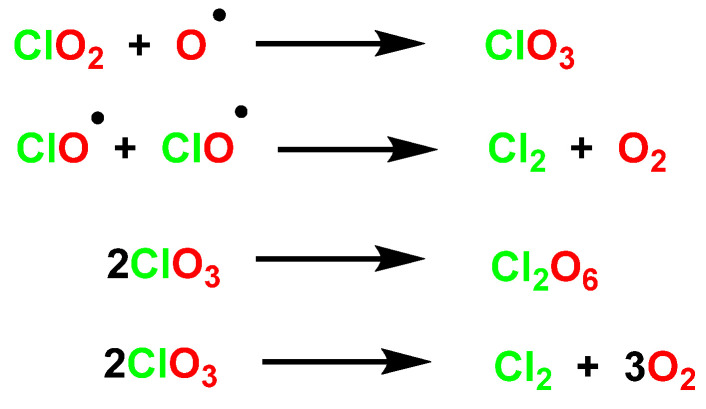
The photolysis of dry, gaseous ClO_2_.

Once homolytic fission has occurred, further reactions will depend on the reaction conditions. At room temperature, photolysis of dry, gaseous ClO_2_ gives Cl_2_, O_2_, and some ClO_3_, which subsequently dimerizes to Cl_2_O_6_ or undergoes further photolysis to Cl_2_ and O_2_, Figure 20.

**Figure 21:**
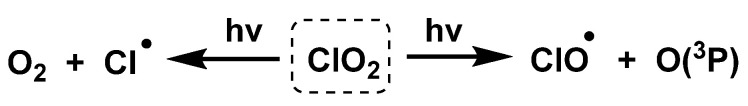
The photochemistry and radical chemistry of photolysis of ClO_2_ by UVC light.

The degradation mechanisms and radical chemistry associated with UVC photolysis of ClO_2_ are quite complicated [51]. The photolysis of ClO_2_ by UVC light provides ClO^−^ and oxygen by cleavage of the Cl-O [52] and Cl^•^ [53] bond, Figure 21.

**Figure 22:**
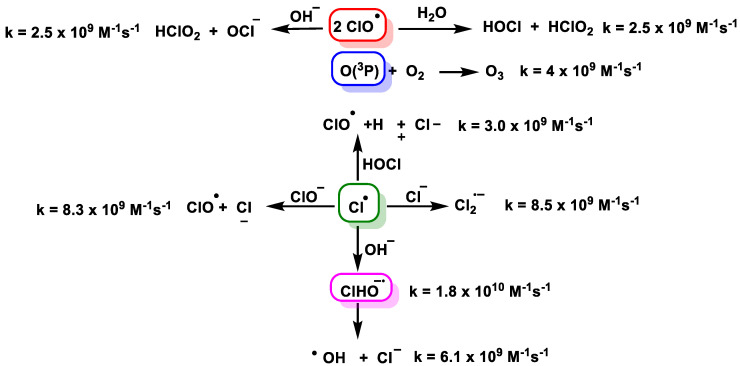
The generated ClO^•^, O(3P), and Cl^•^ undergo chain reactions to generate secondary reactive species.

The above species can undergo chain reactions to generate secondary reactive species [54,55], Figure 22.

**Figure 23:**
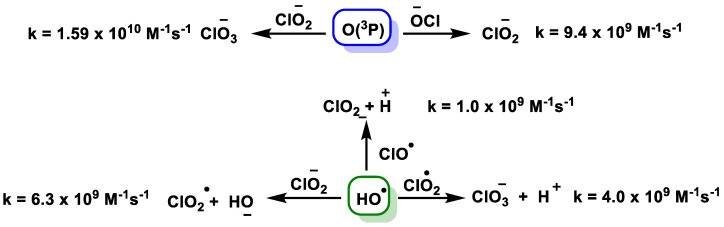
Chlorite and chlorate from the radical-radical interactions.

The degradation of ClO_2_ under UVC radiation accelerates the tendency of chlorite and chlorate formation compared to ClO_2_ alone. In addition, chlorite and chlorate can also be generated from radical-radical interactions [56,57,58,59], Figure 23.

## 5. Reactivity of ClO_2_

The chemistry of ClO_2_ is complex compared to that of other chlorine compounds, because of its high reactivity. Chlorine dioxide is a strong oxidizing agent and, unlike chlorine, does not tend to react with organic materials to form chlorinated species, or with ammonia to form chloramine. The oxidation of ClO_2_ generally begins with the removal of an electron from residual organic compounds to produce organic radicals and ClO_2_^−^. Subsequent oxidation of the organic radicals by ClO_2_ involves oxygen transfer with the release of HOCl or electron transfer with the release of ClO_2_^−^ [46,50].

Inorganic compounds are important in the body and are responsible for many simple functions. The major inorganic compounds are H_2_O, molecular oxygen O_2_, carbon dioxide CO_2_, and some acids, bases, and salts. Iron is a biologically essential component of every living organism and various cellular mechanisms have evolved to capture iron from the environment in biologically useful forms [60]. It is primarily involved in the transfer of oxygen from the lungs to tissues. However, iron also plays a role in metabolism as a component of some proteins and enzymes. Manganese (Mn) is a trace mineral that is present in tiny amounts in the body. It is found mostly in bones, the liver, kidneys, and pancreas, and helps the body form connective tissue, bones, blood clotting factors, and sex hormones. Manganese is a cofactor for many enzymes, including manganese superoxide dismutase, arginase, and pyruvate carboxylase. In these enzymes, manganese is involved in the metabolism of amino acids, cholesterol, glucose, and carbohydrates; the elimination of reactive oxygen species; bone formation; reproduction; immune response; and blood coagulation and hemostasis together with vitamin K [61,62,63,64,65,66,67].

Some researchers have studied the reactivity of ClO_2_ with inorganic and organic compounds has been studied [68]. In the human body, ClO_2_ can react with I^−^, NO_2_^−^, O_3_, H_2_O_2_, Fe(II), and Mn(II). The rate constants with tertiary amines and phenols were also high at pH ≥ 6. ClO_2_ does not react with ammonia, Br^−^, carbohydrates, aromatic hydro-carbides, and compounds containing C=C double bonds at neutral pH conditions.

### 5.1. Reactivity of ClO_2_ with Inorganic Compounds

**Figure 24:**
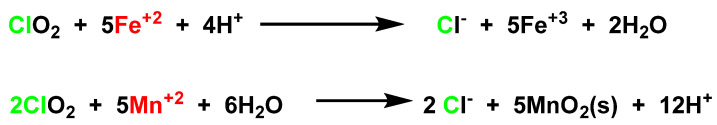
ClO_2_ oxidizes Fe(II) and Mn(II) via rapid one-electron transfer.

ClO_2_ can oxidize many inorganic compounds, being first reduced to chlorite by the transfer of a single electron. In addition, chlorite can react with Fe(II) and Mn(II) [69,70,71,72]; the reactions [70,71] are summarized in Figure 24.

**Figure 25:**
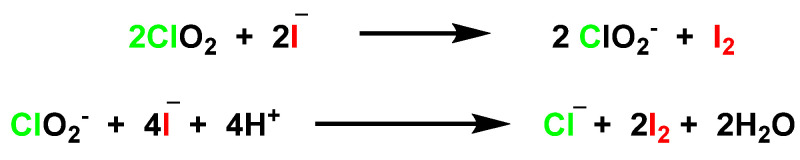
ClO_2_ oxidizes to I^−^ to I_2_.

The reaction rate constants of the ClO_2_ oxidation of Fe(II) and Mn(II) increase greatly with alkaline pH. Iodide, unlike bromide, is readily oxidized in the presence of ClO_2_ to iodine. During oxidation of aqueous iodide, ClO_2_ can rapidly oxidize I^−^ to I_2_ [73]. Chlorite [74,75] produced by the reduction of ClO_2_ can also react with excess I^−^ to form I_2_ at pH 4–8 [76,77,78] (Figure 25).

**Figure 26:**
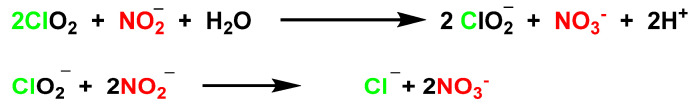
Oxidation of nitrite NO_2_^−^ by ClO_2_.

The nitrite ion is oxidized to nitrate in the presence of ClO_2_. Like iodide, the oxidation of nitrite (NO_2_^−^) by ClO_2_ involves mainly electron transfer reactions (Figure 26).

**Figure 27:**
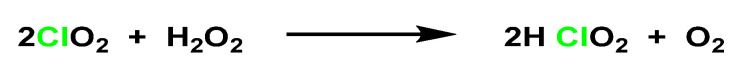
Reaction of hydrogen peroxide with chlorine dioxide.

Chlorite can be further reduced to chloride through reactions with CN^−^ and NO_2_^−^. In the following reaction shown in Figure 27, H_2_O_2_ acts as a reducing agent [8].

**Figure 28:**
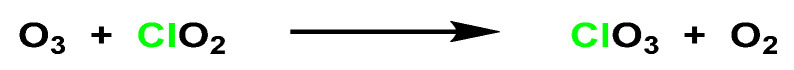
Reaction of ozone with ClO_2_.

In the reaction with O_3_, ClO_2_ is the reducing agent (Figure 28).

### 5.2. Reactivity with Organic Compounds

The reactions of ClO_2_ with organic compounds have generally been investigated in aqueous solutions with low reagent concentrations, in which it reacts with humic and fulvic acids present in water, forming oxidized organic compounds, such as aldehydes and carboxylic acids. It does not form chlorinated organic by-products unless free chlorine is present together with chlorine dioxide.

ClO_2_ reacts with phenolic groups, sulfur compounds, and to a lesser extent, tertiary amines and aromatic amines, while the reaction with hydrocarbons is practically nil. The reactivity of the phenoxide ion and the neutral form of the amine is much greater (by several orders of magnitude) than the reactivity of the neutral form of the phenol and the protonated amine. ClO_2_ tends to react with organic compounds as an electron acceptor and is reduced to chlorite. This makes ClO_2_ a selective oxidant whose reactivity generally favors organic molecules with a lone pair of electrons.

### 5.3. Reactivity with Phenolic Compounds

Chlorine dioxide oxidizes phenolic compounds and has been used to oxidize chlorinated phenolic compounds to reduce their toxicity. At neutral pH, phenols react with ClO_2_ with values between 10^3^–10^8^ M^−1^ s^−1^. The reaction rate constants of phenols dissociated with ClO_2_ are generally six orders of magnitude higher than those of undissociated phenols [79] (Table 1). Therefore, at high pH, the oxidation of phenols with ClO_2_ is favored. The substituents of phenols greatly affect their oxidation rates with ClO_2_.

**Figure 29:**
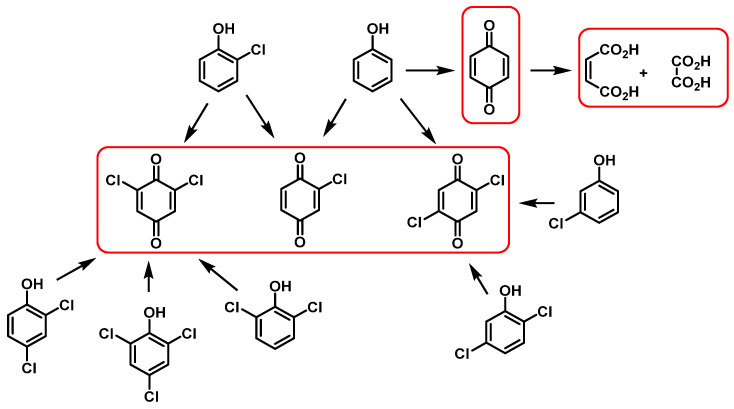
Oxidation of phenols and chlorophenols by reacting with ClO_2_. In red, end products.

The main products of the oxidation of phenols with chlorine dioxide are p-benzoquinone and various substituted chloro-p-benzoquinones [80]. The chlorophenols are oxidized to the corresponding quinones. With a large excess of ClO_2_, the p-quinone is oxidized with ring cleavage, forming dicarboxylic acids. Oxidation of phenols and chloro-phenols [81] is shown in Figure 29.

**Figure 30:**
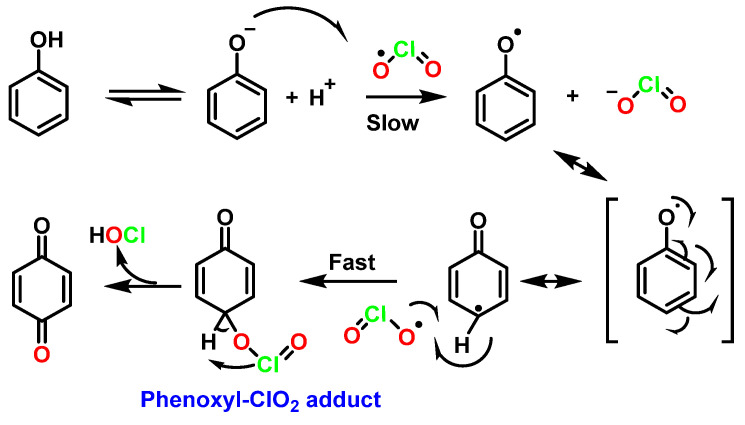
Mechanism of oxidation of phenols with ClO_2_.

This is a two-step mechanism: ClO_2_ reacts with a phenoxide ion that is stabilized to ClO_2_^−^ and a phenoxy radical. This radical reacts rapidly with a second equivalent of ClO_2_ to produce p-benzoquinone and release HOCl. In this mechanism, it was suggested that a phenoxy radical and ClO_2_ radical intermediate could be formed [82] (Figure 30).

**Figure 31:**
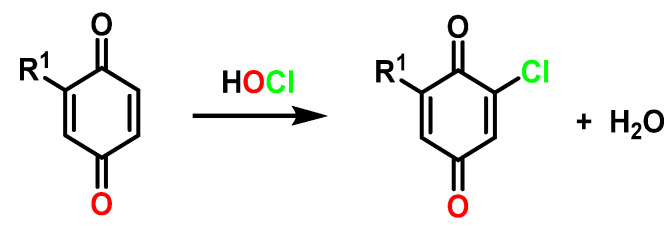
Formation of chloroquinones in the oxidation of phenols with chlorine dioxide.

Chlorinated derivatives in the oxidations of phenols with ClO_2_ can be explained by the hypochlorous acid formed in the reaction [83] (Figure 31).

### 5.4. Reactivity with Amines

#### 5.4.1. Reactivity with Aromatic Amines

Aromatic amines are widely distributed in aqueous media, sometimes as degradation products of herbicides (in agriculture) or dyes in industrial wastewater [84].

**Figure 32:**
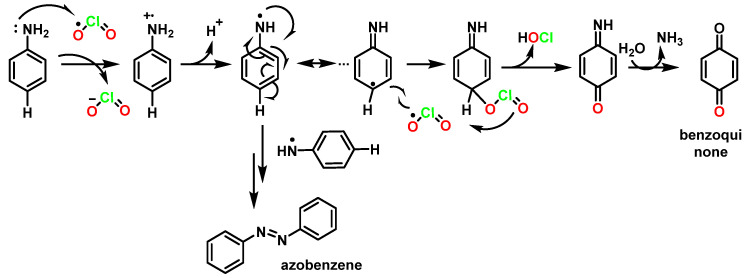
Mechanism for the oxidation of aromatic amines with ClO_2_.

The mechanism of ClO_2_ oxidation of aniline begins with an electron transfer in the first step. The amino group is directly attached to a benzene ring (and is a high electron density center), so there is a change in the electron density of the nitrogen atom as it gives up charge to the benzene ring. The reaction pathways and products obtained are different from those observed with aliphatic amines. The main product obtained is the quinone-azobenzene derivative (Figure 32).

Reaction rates of aniline (4.5 × 10^5^ M^−1^ s^−1^) and two substituted anilines: 4-Aminoaniline (3.5 × 10^8^ M^−1^ s^−1^) and N,N-dimethylaniline ( 4.4 × 10^6^ M^−1^ s^−1^) at pH 7 [75].

#### 5.4.2. Reactivity with Aliphatic Amines

Aliphatic amines are widely distributed in aqueous media, and they react quickly with ClO_2_ to form freely available chlorine FAC [85]. Second-order rate constants for reactions of chlorine dioxide with aliphatic amines in aqueous solutions are listed in Table 2.

Tertiary amines react with ClO_2_ very quickly; secondary and especially primary amines react much more slowly, and ammonia does not react with ClO_2_ at all [80,87].

ClO_2_ oxidizes most aliphatic tertiary amines rapidly and converts them to secondary amines, also forming aldehydes. The possible mechanism is the formation of an aminyl cation radical and chlorite followed by the elimination of a proton at alpha, forming an amine thatsubsequently hydrolyses to the aldehyde and secondary amine [8].

### 5.5. Reactivity with Amino Acids, Peptides, and Proteins

Reaction rates with amines decrease in the order tertiary amine > secondary amine > primary amine. For tertiary amines, the reaction rate constants are in the range 10^3^–10^6^ M^−1^ s^−1^ at neutral pH and are between 2–5 orders of magnitude higher than for secondary or primary amines. ClO_2_ reacts much faster with deprotonated amines than with neutral species because deprotonated amines are stronger electron donors [90,91].

The reactivity of ClO_2_ with biologically important molecules (including amino acids and some peptides) has been well studied [92,93]. ClO_2_ is an effective and promising alternative to other chlorine-containing disinfectants, and a thorough understanding of the chemistry of interactions with amino acids, proteins, and peptides is needed.

**Figure 33:**
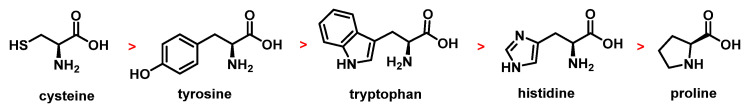
Reactivity of cysteine, tyrosine, tryptophan histidine, and proline with ClO_2_.

**Figure 34:**
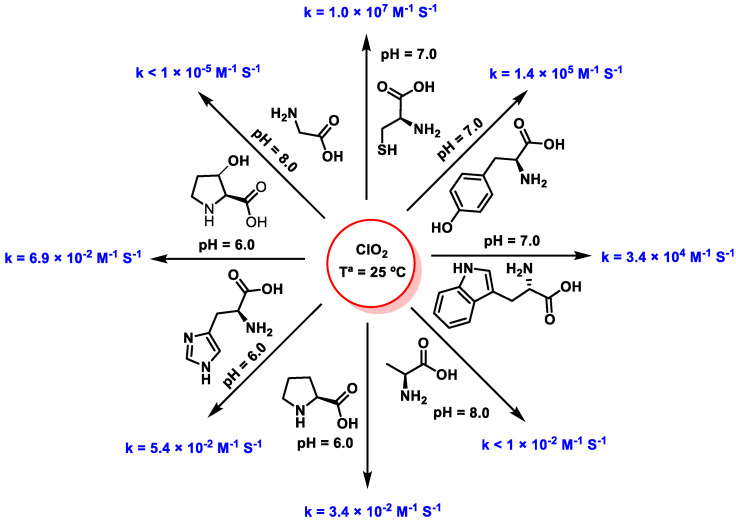
Reaction rate constants of ClO_2_ with amino acids at 25 °C [7].

ClO_2_ reacts rapidly with cysteine, tyrosine, and tryptophan (10^4^–10^7^ M^−1^ s^−1^), but slowly with histidine, proline, alanine, and glycine (10^−5^–10^−2^ M^−1^ s^−1^) [93]. Amino acids have a primary amine in their structure; this amino group is not reactive with ClO_2_. Amino acids reactive with ClO_2_ contain other reactive groups such as phenols or sulfur groups. The following order of reactivity has been reported (Figure 33 and Figure 34).

**Figure 35:**
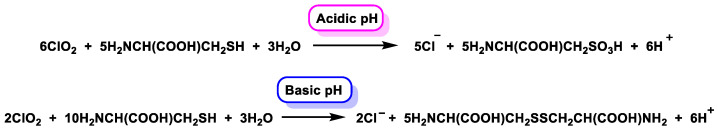
Reaction rate constants of ClO_2_ with amino acids at 25 °C.

Cysteine, due to its nucleophilic -SH group, is the most reactive amino acid with ClO_2_ [94]. Oxidation of cysteine by ClO_2_ has been studied in detail, determining the stoichiometry and reaction products [95]. The stoichiometry of the reaction ([ClO_2_]: [Cys]) was found to be pH-dependent, being 1:0.9 in acidic media and 1:3.7 in basic media (Figure 35).

**Figure 36:**
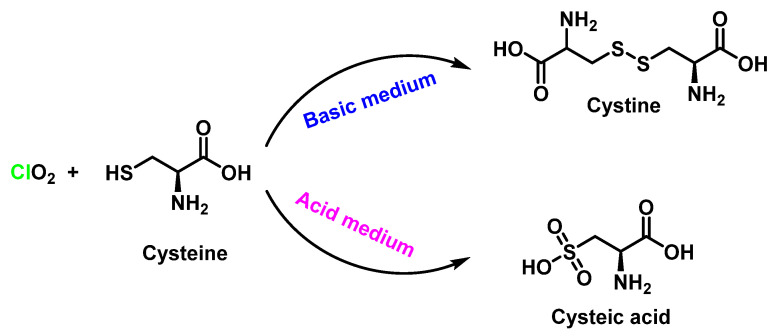
At acidic pH, cysteine sulfonic acid was produced. At alkaline pH, cystine was obtained.

At acidic pH, cysteine sulphonic acid was produced, while at alkaline pH, cystine was obtained, which are products of the oxidation of cysteine by ClO_2_ (Figure 36).

**Figure 37:**
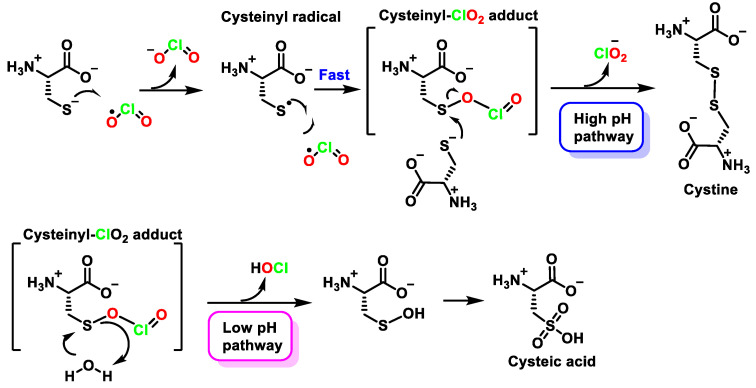
Proposed mechanism for the reactions between ClO_2_ and cysteine.

The reactive cysteine species is the thiolate ion, and it is proposed that the rate-determining step involves electron abstraction from the thiolate ion by ClO_2_ to give the cysteinyl radical. This radical reacts rapidly with another ClO_2_ molecule to form a cysteinyl-ClO_2_ adduct, which is disproportionated by two pH-dependent pathways to produce cystine and cysteic acid (Figure 37).

The reactivity of glutathione is like that of cysteine, and similar steps in the reaction with ClO_2_ are proposed. A study of the oxidation of thiols (Cys and GSH) by ClO_2_ with varying pH has been performed. The rate constant for Cys and GSH increased with pH from 3.2 to 5.9. The pH-dependent behavior suggests that deprotonated thiols are the reactive species. The rate constants [94,95] for the reactions of ClO_2_ with cysteinyl anion (CS^−^) and glutathione anion (GS^−^) are 1.0 × 10^8^ M^−1^ s^−1^ and 1.4 × 10^8^ M^−1^ s^−1^, respectively. Similar rate constants suggest common oxidation mechanisms for cysteine and glutathione by ClO_2_.

In the reactions of histidine, tryptophan, and tyrosine with ClO_2_, different products are obtained depending on the molar ratios of ClO_2_. The products also vary if the reaction is done in the presence or absence of oxygen. With an excess of ClO_2_, low molecular weight compounds are obtained.

**Figure 38:**
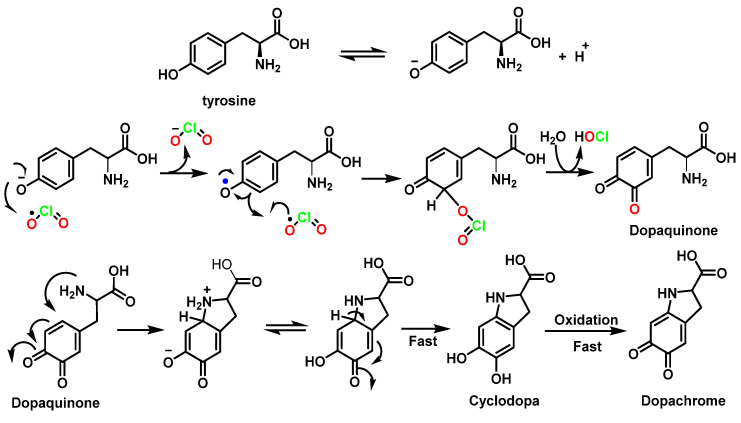
Reaction pathways proposed for ClO_2_ oxidation of the amino acid tyrosine.

ClO_2_ oxidation of tyrosine occurs predominantly in its phenolic structure, resulting in the formation of dopaquinone and dopachrome at pH 6–7. Cyclisation of dopaquinone occurred at pH > 4 to form cyclodopa, which was subsequently oxidized to dopachrome [96] (Figure 38).

**Figure 39:**
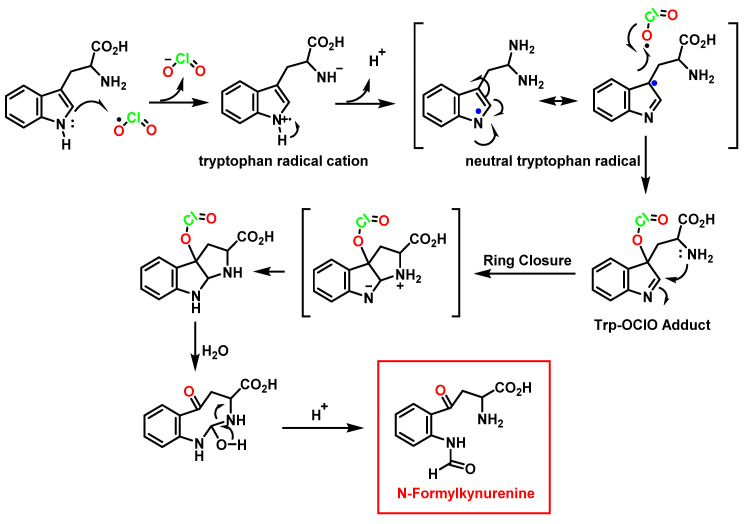
Proposed reaction mechanism for the attack of ClO_2_ to tryptophane.

The product of tryptophan oxidation by ClO_2_ was identified as N-formyl alkyl-nurenine [97]. The initial reaction between tryptophan and ClO_2_ is a one-electron oxidation to form a tryptophan radical cation and a chlorite ion. The radical cation deprotonates to form a neutral tryptophilic radical, which reacts rapidly with a second ClO_2_ molecule to give a short-lived adduct (k_obs_ = 48 s^−1^) with formation of the C-OClO bond. This adduct decomposes to give HOCl [8] (Figure 39).

**Figure 40:**
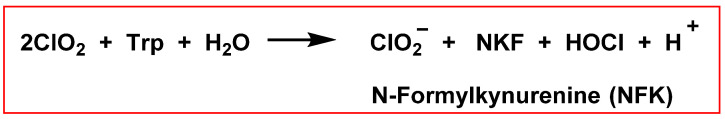
Stoichiometry of the reaction between tryptophan and ClO_2_.

The reaction consumes two ClO_2_ per Trp and forms chlorite and HOCl (Figure 40).

### 5.6. Oxidation of Peptides and Proteins by ClO_2_

ClO_2_ is a selective oxidant that only reacts with five amino acids: cysteine, tyrosine, tryptophan, histidine, and proline. Cysteine, tyrosine, and tryptophan have much faster reaction rate constants. Mass spectrometry and nuclear magnetic resonance spectroscopy show that tryptophan residues are converted to N-formyl alkyl-nurenine and tyrosine residues are converted to 3,4-dihydroxyphenylalanine (DOPA) or 2,4,5-trihydroxyphenylalanine (TOPA) in ClO_2_-treated proteins. Tryptophan residues are critical targets in the reaction between ClO_2_ and proteins [98,99], causing protein fragmentation and denaturation. Inactivation of influenza A virus when treated with ClO_2_ has been observed due to oxidation of a tryptophan residue (W^153^) that was converted to NFK in hemagglutinin, restricting its ability to bind to host cells [100].

Using bovine serum albumin and glucose-6-phosphate dehydrogenase (G6PD) from baker’s yeast (*Saccharomyces cerevisiae*) as model proteins, it was shown that the antimicrobial activity of ClO_2_ is mainly attributed to its protein denaturing activity. Elemental analyses show that oxygen atoms, but not chlorine atoms, are incorporated into the ClO_2_-treated protein, providing direct evidence that ClO_2_ oxidizes the protein. For glutathione, a tripeptide consisting of glycine, cysteine, and glutamic acid, the ClO_2_-reactive site is the thiol group, and the oxidation products are like those of cysteine (Table 3).

### 5.7. Oxidation of NADH by Chlorine Dioxide

**Figure 41:**
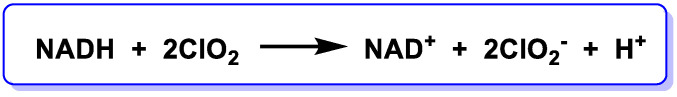
Reaction of NADH with ClO_2_.

The oxidation of dihydronicotinamide adenine dinucleotide (NADH) by chlorine dioxide in phosphate-buffered solutions (pH 6–8) is very fast, with a second-order rate constant of 3.9 × 10^6^ M^−1^ s^−1^ at 24.6 °C. The stoichiometry is shown in Figure 41.

**Figure 42:**
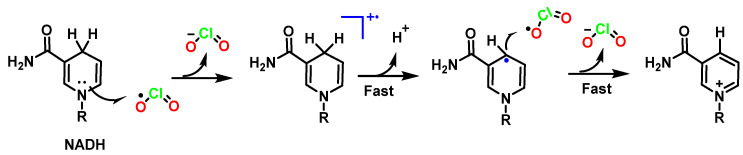
Proposed mechanism of NADH oxidation by ClO_2_.

Unlike many oxidants in which NADH reacts by hydride transfer, the proposed mechanism is a one-electron transfer from NADH to ClO_2_. First, chlorine dioxide accepts an electron from NADH to form ClO_2_^−^ and the radical cation NADH^+^. Then, the subsequent sequence of very rapid deprotonation with the transfer of H^+^ to H_2_O and the transfer of an electron to a second equivalent of ClO_2_ gives as products 2ClO_2_^−^, H_3_O^+^, and NAD+ [101] (Figure 42).

The mechanism by which ClO_2_ influences biomolecules is based on the strong interference with redox processes occurring in mitochondrial and cell membranes, e.g., on the NADH/NAD^+^ system, which is responsible for cellular respiration and for mediating ATP synthesis [102].

## 6. Oxidation of Hemoglobin by ClO_2_

Chlorine dioxide is an oxidizing agent that converts hemoglobin (oxygen-carrying protein) into methemoglobin, which cannot bind to other oxygen molecules and therefore hinders oxygenation of the body. In these cases, as when ingested in large quantities, ClO_2_ oxidizes ferrous iron (Fe^2+^) and transforms it into ferric iron (Fe^3+^), and hemoglobin becomes methemoglobin, which causes respiratory failure [33].

**Figure 43:**
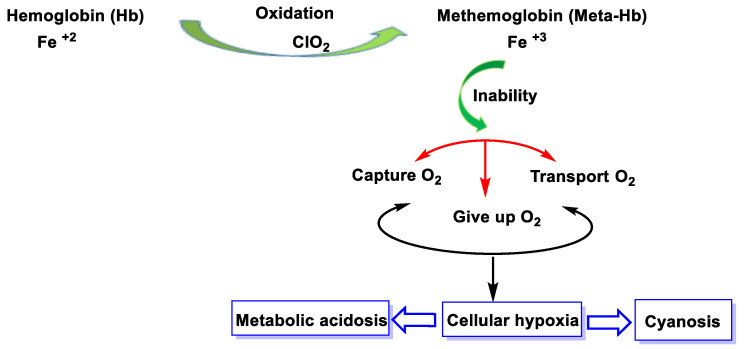
Methemoglobinemia pathology.

Methemoglobin is an oxidized form of hemoglobin that is unable to carry oxygen in the blood and is therefore unable to release it effectively into the body’s tissues, thus preventing oxygenation of the body. High levels of methemoglobin can have other risks. Methemoglobin-forming chemicals can oxidate the ferrous nucleus of hemoglobin (Fe^2+^) into trivalent iron (Fe^3+^), transforming hemoglobin into methemoglobin. Its toxic effects are due to the reduced oxygen-carrying capacity of methemoglobin, resulting in cellular hypoxia [103,104] (Figure 43).

In 2015, the first case of a child with methemoglobinemia (high methemoglobin levels) after accidentally ingesting chlorine dioxide appeared in the literature. The authors reported that “the patient had profound hypoxia, did not respond to oxygen therapy, and required endotracheal intubation to maintain a normal oxygen level” [105].

In another publication in 2013, a person who tried to commit suicide and ingested less than 100 mL of a 28% sodium chlorite solution had 40% methemoglobin in his blood, requiring a kidney transplant and transfusions to save his life [106].

For these reasons, specialists conclude that chlorine dioxide not only deoxygenates the body, it can cause low tissue oxygenation capacity even in small doses, a situation that can put people’s lives at risk.

## 7. Toxicity of ClO_2_

In December 2019, a new respiratory illness emerged in Wuhan, China. The source of this infection was identified as a new coronavirus, related to other coronaviruses that had previously caused outbreaks of SARS (Severe Acute Respiratory Syndrome) between 2002 and 2004 and MERS (Middle East Respiratory Syndrome) in 2012 (National Institutes of Health, 2020). This virus was named “severe acute respiratory syndrome coronavirus 2” (SARS-CoV-2) and the disease resulting from infection with this virus was named “COVID-19”. On 11 March 2020, the World Health Organization WHO declared COVID-19 a pandemic. Coronaviruses are a group of enveloped RNA viruses that can damage multiple organ systems. Like other coronaviruses, SARS-CoV-2 is a spherical particle with glycoprotein spikes on its surface. Coronaviruses enter host cells when a region of the spike, known as the “receptor-binding domain”, binds to angiotensin-converting enzyme 2 (hACE2) in human cells. The viral membrane then fuses with the host cell membrane, allowing the viral genome to enter the host cell.

During the COVID-19 pandemic, the consumption of chlorine dioxide solutions has been promoted through different avenues (social networks, websites, mass media) for the treatment or prevention of SARS-CoV-2 infection. Different regulatory agencies (such as the European Medicines Agency and the US Food and Drug Administration) and scientific societies have drafted and issued statements warning about the lack of scientific evidence for their efficacy in COVID-19 disease and the associated risks to human health, and even demanded the withdrawal of these products from the market.

The FDA (Food and Drug Administration) in the United States of America and COFEPRIS (Comisión Federal para la Protección contra Riesgos Sanitarios) in Mexico state that the consumption of ClO_2_ causes kidney and liver failure and destroys red blood cells. To date, there is no scientific evidence to support the use of chlorine dioxide or chlorine derivatives as preventive or therapeutic agents against COVID-19 [107,108,109].

**Figure 44:**
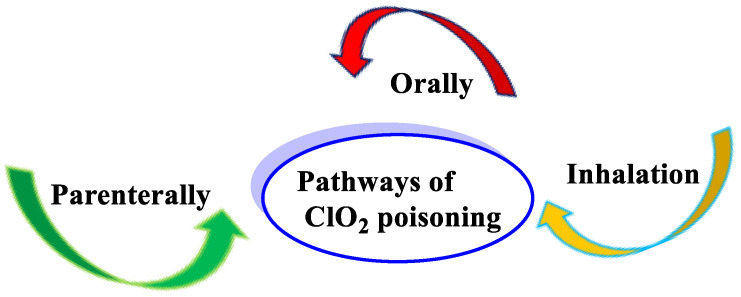
The main routes of ClO_2_ poisoning.

Studies have described the toxic effects of chlorine dioxide ingestion. The main routes of intoxication can be divided into three: inhalation, oral, and parenteral routes (Figure 44).

Chlorine dioxide can be rapidly absorbed through the gastrointestinal tract. Peak blood concentration levels can be reached within 1 h after a single dose administered orally. It can also be absorbed slowly through shaved skin with a median absorption time of 22 h. Intact chlorine dioxide is unlikely to be absorbed by inhalation given its highly reactive nature; it is more likely that its derivatives can be absorbed. Chlorine dioxide is metabolized to chlorite, chlorate, and mainly chloride. Most of the administered chlorine dioxide and its metabolites remain in the plasma, followed by the kidneys, lungs, stomach, intestine, liver, and spleen. About 43% of orally administered chlorine dioxide is excreted in the urine and feces within 72 h.

It is important to note that neither chlorine dioxide nor its derivatives have undergone any evaluation or authorization by the competent authorities to ensure that the benefit/risk ratio is positive for the population.

**Figure 45:**
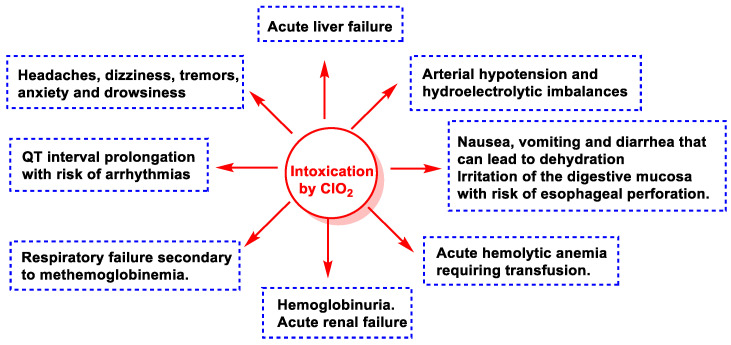
Serious adverse reactions after direct consumption of chlorine dioxide.

There is no published scientific evidence that has positively considered the use of chlorine dioxide or its derivatives as a preventive or therapeutic agent against COVID-19 administered by inhalation, oral, or parenteral routes [109,110]. Some of the risks of consuming ClO_2_ and its derivatives are listed in Figure 45.

The median oral lethal dose (LD50) has been estimated to be 94 mg/kg body weight and it is therefore considered a moderately toxic and hazardous substance. The Spanish Agency for Medicines and Health Products (AEMPS) warns of serious health risks from the consumption of chlorine dioxide [111].

## 8. Antimicrobial Activity of ClO_2_

Chlorine dioxide acts as an oxidizing biocide and controls the growth of Gram-positive and Gram-negative bacteria by inhibiting the transport of nutrients through the cell wall by destroying it [112]. Its effectiveness is similar or even superior in some respects to that of other known oxidants such as ozone or chlorine. It behaves as an oxidizing agent through electronic exchange, which allows it to oxidize any type of organic compound, from viruses and bacteria to proteins, hence its frequent use to purify water or certain surfaces. Pereira et al., 2008, compared the efficacy of HOCl, ClO_2_, and O_3_ in the inactivation of *Cryptosporidium oocyst* in a public water supply from Brazilian South conditions. Experiments were carried out in samples containing 2 × 10^4^ oocysts/mL of *C. parvum* purified from feces of experimentally contaminated calves. By using HOCl, the maximum inactivation rate obtained was 49.04% after 120 min, at 2 ppm. ClO_2_ at 5 ppm inactivated 90.56% of oocysts after 90 min of contact. O_3_ was the most effective product, rendering an inactivation of 100% at 24 ppm.

In the case of enveloped viruses, chlorine dioxide reacts directly with amino acid residues of proteins located on the enveloped viral surface; in the case of non-enveloped viruses, ClO_2_ acts on the viral genome, affecting the ribonucleic acid RNA in the cell. By this mechanism, chlorine dioxide prevents the production of proteins and, therefore, promotes the elimination of the virus. Chlorine dioxide is a strong oxidizing agent that can be applied both in solution and in a gaseous state. It has bactericidal, fungicidal, and virucidal properties. Several food-related microorganisms, including Gram-negative and Gram-positive bacteria, yeasts and mold spores, and *Bacillus cereus* spores, were tested for susceptibility to 0.08 mg/L gaseous ClO_2_ for 1 min at 90% relative humidity [17]. In this screening, according to Vandekinderen et al., 2009, the resistance of the different groups of microorganisms to gaseous ClO_2_ generally increased in the order of Gram-negative bacteria, Gram-positive bacteria, yeast spores, molds, and *Bacillus cereus* spores. Factors influencing the antimicrobial efficacy of gaseous ClO_2_ were its concentration, contact time, relative humidity, and temperature. Yeasts were more resistant to ClO_2_ than Gram-negative and Gram-positive bacteria. Significantly, ClO_2_ has been shown to be effective in inactivating *Bacillus anthracis* spores [113,114,115] in government and commercial buildings; however, *Bacillus cereus* was little affected by ClO_2_ [113,114,115,116].

**Figure 46:**
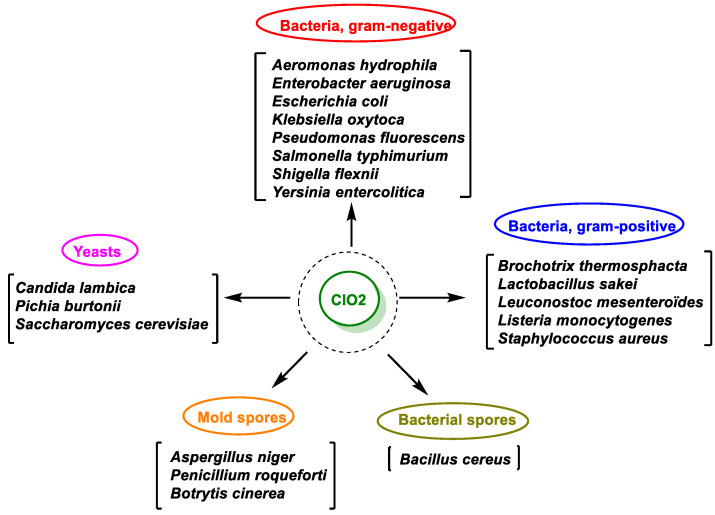
ClO_2_ antimicrobial spectrum of activity.

The resistance of different groups of microorganisms to gaseous ClO_2_ generally increased in the order of Gram-negative bacteria, Gram-positive bacteria, yeast and mold spores, and *Bacillus cereus* spores. ClO_2_ arguably provides the complete solution for disinfection because it kills the widest variety of microbes in short contact times and has fewer corrosive effects on surfaces. In addition, the use of ClO_2_ avoids the threat of microbial resistance (Figure 46).

**Figure 47:**
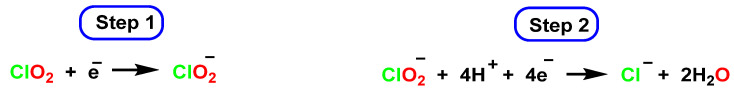
Reduction of chlorine dioxide.

The oxidative capacity of chemicals denotes the number of electrons a molecule can accept from surrounding molecules. In the case of ClO_2_, it can gain five electrons from microbial species per molecule, making it a superior biocide to alternative oxidants, which can normally only gain two. This enhanced effect is attributed to its two-step reduction (Figure 47).

Figure 41 shows the reduction of ClO_2_. In the first step, ClO_2_ is reduced to chlorite after accepting one electron and then further reduced by accepting four additional electrons and four hydrogen atoms [117]. This two-step process allows it to sequester a greater number of electrons from microbes compared to other oxidants. This means that chlorine dioxide will have a reduced corrosive effect on the surfaces to which it is applied, while having a greater ability to kill. The reason why oxidizing agents such as ClO_2_ are preferred to non-oxidizing disinfectants is due to their proven efficacy against bacterial spores and other microorganisms in short contact times.

Chlorine dioxide kills pathogens through electron exchange, sequestering electrons from the microorganism’s structures, such as cell walls, membranes, organelles, and genetic materials, causing a molecular imbalance that leads to the death of the microorganism. Microbes cannot develop resistance to ClO_2_ due to the reaction mechanism and are destroyed.

Biocides, such as quaternary ammonium compounds and triamines, contribute to increased microbial resistance, and several resistant strains, such as *E. coli* and *C. difficile* spores, have been identified. In contrast, microbial resistance is not possible with ClO_2_ because of its mode of action, which is modifying microbial structures and targeting their physiological molecular integrity. This induces membrane rupture, disrupting protein function, inhibiting RNA synthesis, and killing the microbes.

## 9. Conclusions

In this study, the research has focused on two complementary aspects: (i) on analyzing the reactivity of ClO_2_ and its possible reactions with organic and inorganic compounds; and (ii) its potential uses and its toxicity if consumed out of specification.

ClO_2_ is added to drinking water to protect people from harmful bacteria and other microorganisms. The Environmental Protection Agency (EPA) recognizes chlorine dioxide use as a drinking water disinfectant, and it is included in WHO’s Guidelines for drinking-water quality. When added to drinking water, it helps destroy bacteria, viruses, and some types of parasites that can make people sick, such as *Cryptosporidium parvum* and *Giardia lamblia*. EPA regulates the maximum concentration of chlorine dioxide in drinking water to be no greater than 0.8 ppm. In medical settings, ClO_2_ can be used to help sterilize equipment, surfaces, rooms, and tools. In hospitals and other healthcare environments, ClO_2_ helps to sterilize medical and laboratory equipment, surfaces, rooms, and tools. Researchers have found that at appropriate concentrations, ClO_2_ is both safe and effective at helping to eliminate *Legionella* bacteria in hospital environments. *Legionella pneumophila* bacteria can cause Legionnaires’ disease, a potentially deadly type of pneumonia [118,119].

ClO_2_ is highly reactive, reacting to oxidize inorganic and organic compounds found in water, including humic and fulvic acids, forming oxidized organic compounds such as aldehydes and carboxylic acids. Inside cells, ClO_2_ oxidizes phenolic compounds, amines, amino acids, peptides, and proteins, as well as NADH, whose key function is to regulate electron and proton exchange and energy production in all cells. Their effect on biomolecules arises from interference with redox processes, modifying the electronic exchanges that occur in complexes I-IV of mitochondrial respiration and cell membranes.

Depending on concentration and frequency, it is toxic to human health, hence there are limits to its exposure to ensure safe use. The mean oral lethal dose LD50 for rats is 94 mg per kg body weight; it is therefore classified as a moderately toxic and hazardous substance. According to the classification provided by companies to the European Chemical Agency (ECHA) in the REACH registrations, this substance is fatal by inhalation, toxic by ingestion, causes severe skin burns and eye damage, and is very toxic to the environment and aquatic life, with long-lasting effects.

During the COVID-19 pandemic, the consumption of ClO_2_ solutions has been promoted by non-scientists and non-medical people through different avenues (social networks, websites, mass media) for the treatment or prevention of SARS-CoV-2 infection. To date, there is no scientific evidence to uphold the use of ClO_2_ or chlorine derivatives as preventive or therapeutic agents against COVID-19. Its action is unproven, and deaths have been reported, so health agencies such as the US Food and Drug Administration (FDA) have officially stated that they do not recommend taking it. Some of the common symptoms of intoxication include severe vomiting and diarrhea, anemia, severe liver failure, low blood pressure, arrythmia, and methemoglobinemia [120].

Ingestion of ClO_2_ outside the regulations approved by health authorities can have serious results, including intestinal perforation. It is important to emphasize the need to follow communications and warnings from health authorities and governmental institutions. There are documented cases, both in the scientific literature and in the popular media, of severe side effects caused by ClO_2_ poisoning. According to court documents, in the US alone, poison control centers have treated more than 16,000 cases of chlorine dioxide poisoning from 2014 to the end of 2020 [121].

## Figures and Tables

**Table 1:** Constants determined for ClO_2_ reactions with phenols.

Compound	Solvent	pH	T °C	K (M^−1^ s^−1^ )
Phenoxide ion	H_2_O		23	4.9 × 10^7^
Phenol	H_2_O			0.24
2-Chlorophenoxide ion	H_2_O	2–5	23	3.5 x 10^7^
2-Chlorophenol	H_2_O	2–5	23	1.5

**Table 2:** Second-order rate constants for reactions of chlorine dioxide with aliphatic amines.

Compound	pH	T °C	K (M^−1^ s^−1^ )	Refs
Benzylamine	8.96	25.0	4.1 × 10^−2^	[86]
Benzyl-*tert*-butylamine	8.4	25.0	2.9 × 10^2^	[86]
N,N- dimethy-3-methoxybenzylamine		27.0	2.9 × 10^4^	[87]
Methylamine	7–10	25.0	<1	[85]
Dimethylamine	6.8–9.3	24.0	5 × 10^2^	[88]
Trimethylamine		23.0	6 × 10^4^	[89]

**Table 3:** Constants determined for ClO_2_ reactions with peptides and proteins.

Compound	pH	T °C	K (M^−1^ s^−1^ )
**Peptides**			
Glutathione	5.9	25.0	1.4 × 10^8^
**Proteins**			
Bovine serum albumin	7.0	25.0	6.4
Glucosa-6-fosfato deshidrogenasa	7.0	25.0	9.7

## Data Availability

Not applicable.

## References

[1] Aieta E.M., Berg J.D. (1986). A Review of Chlorine Dioxide in Drinking Water Treatment. J. (Am. Water Work. Assoc.).

[2] Deshwal B.R., Lee H.-K. (2005). Manufacture of Chlorine Dioxide from Sodium Chlorate: State of the Art. J. Ind. Eng. Chem..

[3] Mei L., Shilong T., Jin S., Xizhuo W., Jianxin C., Shouqiang L., Xia G., Jiachun T. (2017). Effects of chlorine dioxide on morphology and ultrastructure of Fusarium sulphureum and its virulence to potato tubers. Int. J. Agric. Biol. Eng..

[4] Wang Y., Liu H., Xie Y., Ni T., Liu G. (2015). Oxidative removal of diclofenac by chlorine dioxide: Reaction kinetics and mechanism. Chem. Eng. J..

[5] Friedline A.W., Zachariah M.M., Middaugh A.N., Heiser M.J., Khanna N., Vaishampayan P., Rice C.V. (2015). Sterilization of hydrogen peroxide resistant bacterial spores with stabilized chlorine dioxide. AMB Express.

[6] Al-Hamzah A., Rahman M.M., Kurup P.K., Barnawi A., Ghannam B., Musharraf I., Najjar F.A., Obeidallah A., Palmer N. (2019). Use of chlorine dioxide as alternative to chlorination in reverse osmosis product water. Desalinat. Water Treat..

[7] Zhang J.-Y., Zhang X., Chen J.-H., Deng C.-H., Xu N., Shi W., Cheng P. (2016). Highly selective luminescent sensing of xylene isomers by a water stable Zn-organic framework. Inorg. Chem. Commun..

[8] Gan W., Ge Y., Zhong Y.-H., Yang X. (2020). The reactions of chlorine dioxide with inorganic and organic compounds in water treatment: Kinetics and mechanisms. Environ. Sci. Water Res. Technol..

[9] Jeong C.H., Wagner E.D., Siebert V.R., Anduri S., Richardson S.D., Daiber E.J., Mckague A.B., Kogevinas M., Villanueva C.M., Goslan E.H. (2012). Occurrence and toxicity of disinfection byproducts in European drinking waters in relation with the HIWATE epidemiology study. Environ. Sci. Technol..

[10] Sharma V.K., Sohn M. (2012). Oxidation of Amino Acids, Peptides, and Proteins by Chlorine Dioxide. Implications for Water Treatment. Environmental chemistry for a sustainable world.

[11] Tsai L.S., Higby R., Schade J.E. (1995). Disinfection of Poultry Chiller Water with Chlorine Dioxide: Consumption and Byproduct Formation. J. Agric. Food Chem..

[12] Kim J., Huang T.-S., Marshall M.R., Wei C.I. (1999). Chlorine Dioxide Treatment of Seafoods to Reduce Bacterial Loads. J. Food Sci..

[13] Kim H., Kang Y., Beuchat L.R., Ryu J.-H. (2008). Production and stability of chlorine dioxide in organic acid solutions as affected by pH, type of acid, and concentration of sodium chlorite, and its effectiveness in inactivating Bacillus cereus spores. Food Microbiol..

[14] Kull T.P.J., Backlund P.H., Karlsson K.M., Meriluoto J. (2004). Oxidation of the cyanobacterial hepatotoxin microcystin-LR by chlorine dioxide: Reaction kinetics, characterization, and toxicity of reaction products. Environ. Sci. Technol..

[15] Kull T.P.J., Sjövall O., Tammenkoski M., Backlund P.H., Meriluoto J. (2006). Oxidation of the cyanobacterial hepatotoxin microcystin-LR by chlorine dioxide: Influence of natural organic matter. Environ. Sci. Technol..

[16] Gordon G., Rosenblatt A.A. (2005). Chlorine Dioxide: The Current State of the Art. Ozone Sci. Eng..

[17] Vandekinderen I., Devlieghere F., Van Camp J., Kerkaert B., Cucu T., Ragaert P., De Bruyne J., De Meulenaer B. (2009). Effects of food composition on the inactivation of foodborne microorganisms by chlorine dioxide. Int. J. Food Microbiol..

[18] Taylor J., Wohlers D.W., Amata R. (2004). Toxicological Profile for Chlorine Dioxide and Chlorite. Draft for Public Comment.

[19] Vogt H., Balej J., Bennett J.E., Wintzer P., Sheikh S.A., Gallone P., Vasudevan S., Pelin K. (2010). Chlorine Oxides and Chlorine Oxygen Acids. https://onlinelibrary.wiley.com/doi/abs/10.1002/14356007.a06_483.

[20] Bastidas-Ortiz (2021). Chlorine Dioxide: Does It Contribute to Human Health? A Brief Review. https://www.truthforhealth.org/2022/06/chlorine-dioxide-does-it-contribute-to-human-health-a-brief-review/.

[21] Jin R., Hu S.-Q., Zhang Y.-G., Bo T. (2009). Concentration-dependence of the explosion characteristics of chlorine dioxide gas. J. Hazard. Mater..

[22] Tanaka K., Tanaka T. (1983). CO_2_ and N_2_O laser Stark spectroscopy of the ν1 band of the ClO_2_ radical. J. Mol. Spectrosc..

[23] Brockway L.O. (1933). The Three-Electron Bond in Chlorine Dioxide. Proc. Natl. Acad. Sci. USA.

[24] Jost W. (1954). Linus Pauling: “General Chemistry”.

[25] Marcon J., Mortha G., Marlin N., Molton F., Duboc C., Burnet A. (2017). New insights into the decomposition mechanism of chlorine dioxide at alkaline pH. Holzforschung.

[26] Svenson D.R., Kadla J.F., Chang H.m., Jameel H. (2002). Effect of pH on the Inorganic Species Involved in a Chlorine Dioxide Reaction System. Ind. Eng. Chem. Res..

[27] White G.C. (1992). The Handbook of Chlorination and Alternative Disinfectants.

[28] Tsai Y.-T., Chang C.Y., Hsieh Y.H. (2013). The Generation of Chlorine Dioxide by Electrochemistry Technology. Adv. Sci. Lett..

[29] Pillai K.C., Kwon T.O., Park B.B., Moon I.S. (2009). Studies on process parameters for chlorine dioxide production using IrO_2_ anode in an un-divided electrochemical cell. J. Hazard. Mater..

[30] Brito C.d.N., Araújo D.M.d., Martínez-Huitle C.A.A., Rodrigo M.A. (2015). Understanding active chlorine species production using boron doped diamond films with lower and higher sp3/sp2 ratio. Electrochem. Commun..

[31] Mostafa E., Reinsberg P.H., Garcia-Segura S., Baltruschat H. (2018). Chlorine species evolution during electrochlorination on boron-doped diamond anodes: In-situ electrogeneration of Cl_2_, Cl_2_O and ClO_2_. Electrochim. Acta.

[32] Souza F.L., Sáez C., Lanza M.R.V., Cañizares P., Rodrigo M.A. (2016). The effect of the sp3/sp2 carbon ratio on the electrochemical oxidation of 2,4-D with p-Si BDD anodes. Electrochim. Acta.

[33] Umile T.P., Groves J.T. (2011). Catalytic generation of chlorine dioxide from chlorite using a water-soluble manganese porphyrin. Angew. Chem..

[34] Umile T.P., Wang D., Groves J.T. (2011). Dissection of the mechanism of manganese porphyrin-catalyzed chlorine dioxide generation. Inorg. Chem..

[35] Hicks S.D., Petersen J., Bougher C.J., Abu-Omar M.M. (2011). Chlorite dismutation to chlorine dioxide catalyzed by a water-soluble manganese porphyrin. Angew. Chem..

[36] Zdilla M.J., Lee A.Q., Abu-Omar M.M. (2008). Bioinspired dismutation of chlorite to dioxygen and chloride catalyzed by a water-soluble iron porphyrin. Angew. Chem..

[37] Qi M., Yi T., Mo Q., Huang L., Zhao H., Xu H., Huang C., Wang S., Liu Y., Hui Z. (2020). Preparation of High-Purity Chlorine Dioxide by Combined Reduction. Chem. Eng. Technol..

[38] Monteiro M.K.S., Monteiro M.M.S., de Melo Henrique A.M., Llanos J., Saez C., Dos Santos E.V., Rodrigo M.A. (2021). A review on the electrochemical production of chlorine dioxide from chlorates and hydrogen peroxide. Curr. Opin. Electrochem..

[39] Romero-Fierro D., Bustamante-Torres M., Hidalgo-Bonilla S., Bucio E. (2021). Microbial degradation of disinfectants. Recent Advances in Microbial Degradation.

[40] Guggenberger M., Hettegger H., Zwirchmayr N.S., Hosoya T., Bacher M., Zaccaron S., Böhmdorfer S., Reiter H., Spitzbart M., Dietz T. (2020). Degradation of the cellulosic key chromophore 2, 5-dihydroxy-[1,4]-benzoquinone (DHBQ) under conditions of chlorine dioxide pulp bleaching: Formation of rhodizonate as secondary chromophore—A combined experimental and theoretical study. Cellulose.

[41] Kucera J. (2019). Biofouling of polyamide membranes: Fouling mechanisms, current mitigation and cleaning strategies, and future prospects. Membranes.

[42] Odeh I.N., Francisco J.S., Margerum D.W. (2002). New pathways for chlorine dioxide decomposition in basic solution. Inorg. Chem..

[43] Li J., Cassol G.S., Zhao J., Sato Y., Jing B., Zhang Y., Shang C., Yang X., Ao Z., Chen G. (2022). Superfast degradation of micropollutants in water by reactive species generated from the reaction between chlorine dioxide and sulfite. Water Res..

[44] Kishimoto N. (2019). State of the art of UV/chlorine advanced oxidation processes: Their mechanism, byproducts formation, process variation, and applications. J. Water Environ. Technol..

[45] Saini P., Krishnan A., Yadav D., Hazra S., Elias A.J. (2021). External Catalyst-Free Oxidation of Benzyl Halides to Benzoic Acids Using NaOH/TBHP in Water. Asian J. Org. Chem..

[46] Rougé V., Allard S., Croué J.-P., von Gunten U. (2018). In situ formation of free chlorine during ClO_2_ treatment: Implications on the formation of disinfection byproducts. Environ. Sci. Technol..

[47] Wang L., Margerum D.W. (2002). Hypohalite ion catalysis of the disproportionation of chlorine dioxide. Inorg. Chem..

[48] Firak D.S., Farkas L., Náfrádi M., Alapi T. (2022). Degradation of chlorinated and hydroxylated intermediates in UVA/ClO_2_ systems: A chlorine-based advanced oxidation process investigation. J. Environ. Chem. Eng..

[49] Philpott M.J., Hayes S.C., Reid P.J. (1998). Femtosecond pump–probe studies of chlorine dioxide photochemistry in water and acetonitrile. Chem. Phys..

[50] Chuang Y.-H., Wu K.-L., Lin W.-C., Shi H.-J. (2022). Photolysis of Chlorine Dioxide under UVA Irradiation: Radical Formation, Application in Treating Micropollutants, Formation of Disinfection Byproducts, and Toxicity under Scenarios Relevant to Potable Reuse and Drinking Water. Environ. Sci. Technol..

[51] Kong Q., Fan M., Yin R., Zhang X., Lei Y., Shang C., Yang X. (2021). Micropollutant abatement and byproduct formation during the co-exposure of chlorine dioxide (ClO_2_) and UVC radiation. J. Hazard. Mater..

[52] Cosson H., Ernst W.R. (1994). Photodecomposition of chlorine dioxide and sodium chlorite in aqueous solution by irradiation with ultraviolet light. Ind. Eng. Chem. Res..

[53] Thøgersen J., Jepsen P.U., Thomsen C., Poulsen J.A., Byberg J., Keiding S. (1997). Femtosecond photolysis of ClO_2_ in aqueous solution. J. Phys. Chem. A.

[54] Fang J., Fu Y., Shang C. (2014). The roles of reactive species in micropollutant degradation in the UV/free chlorine system. Environ. Sci. Technol..

[55] Yin R., Blatchley E.R., Shang C. (2020). UV photolysis of mono-and dichloramine using UV-LEDs as radiation sources: Photodecay rates and radical concentrations. Environ. Sci. Technol..

[56] Buxton G.V., Greenstock C.L., Helman W.P., Ross A.B. (1988). Critical review of rate constants for reactions of hydrated electrons, hydrogen atoms and hydroxyl radicals (⋅ OH/⋅ O− in aqueous solution. J. Phys. Chem. Ref. Data.

[57] Buxton G., Subhani M. (1972). Radiation chemistry and photochemistry of oxychlorine ions. Part 2.—Photodecomposition of aqueous solutions of hypochlorite ions. J. Chem. Soc. Faraday Trans. 1 Phys. Chem. Condens. Phases.

[58] Guo K., Wu Z., Shang C., Yao B., Hou S., Yang X., Song W., Fang J. (2017). Radical chemistry and structural relationships of PPCP degradation by UV/chlorine treatment in simulated drinking water. Environ. Sci. Technol..

[59] Zuo Z., Katsumura Y., Ueda K., Ishigure K. (1997). Reactions between some inorganic radicals and oxychlorides studied by pulse radiolysis and laser photolysis. J. Chem. Soc. Faraday Trans..

[60] Abbaspour N., Hurrell R., Kelishadi R. (2014). Review on iron and its importance for human health. J. Res. Med. Sci..

[61] Ross A.C., Caballero B., Cousins R.J., Tucker K.L. (2020). Modern Nutrition in Health and Disease.

[62] Nielsen F.H. (2012). Manganese, molybdenum, boron, chromium, and other trace elements. Present Knowl. Nutr..

[63] Li L., Yang X. (2018). The essential element manganese, oxidative stress, and metabolic diseases: Links and interactions. Oxidative Med. Cell. Longev..

[64] Russell R., Beard J.L., Cousins R.J., Dunn J.T., Ferland G., Hambidge K., Lynch S., Penland J., Ross A., Stoecker B. (2001). Dietary Reference Intakes for Vitamin A, Vitamin K, Arsenic, Boron, Chromium, Copper, Iodine, Iron, Manganese, Molybdenum, Nickel, Silicon, Vanadium, and Zinc.

[65] Aschner J.L., Aschner M. (2005). Nutritional aspects of manganese homeostasis. Mol. Asp. Med..

[66] Palacios C. (2006). The role of nutrients in bone health, from A to Z. Crit. Rev. Food Sci. Nutr..

[67] Chen P., Bornhorst J., Aschner M.A. (2018). Manganese Metabolism in Humans. Front. Biosci. (Landmark Ed.).

[68] Hoigné J., Bader H. (1994). Kinetics of reactions of chlorine dioxide (OClO) in water—I. Rate constants for inorganic and organic compounds. Water Res..

[69] Van Benschoten J.E., Lin W., Knocke W.R. (1992). Kinetic modeling of manganese (II) oxidation by chlorine dioxide and potassium permanganate. Environ. Sci. Technol..

[70] Wang L., Odeh I.N., Margerum D.W. (2004). Chlorine dioxide reduction by aqueous iron (II) through outer-sphere and inner-sphere electron-transfer pathways. Inorg. Chem..

[71] Henderson R., Carlson K., Gregory D. (2001). The impact of ferrous ion reduction of chlorite ion on drinking water process performance. Water Res..

[72] Chen L., Zhang J., Zheng X. (2016). Coupling technique for deep removal of manganese and iron from potable water. Environ. Eng. Sci..

[73] Fábián I., Gordon G. (1997). The kinetics and mechanism of the chlorine dioxide–iodide ion reaction. Inorg. Chem..

[74] Stanbury D.M., Martinez R., Tseng E., Miller C.E. (1988). Slow electron transfer between main-group species: Oxidation of nitrite by chlorine dioxide. Inorg. Chem..

[75] Huie R.E., Neta P. (1986). Kinetics of one-electron transfer reactions involving chlorine dioxide and nitrogen dioxide. J. Phys. Chem..

[76] Fukutomi H., Gordon G. (1967). Kinetic study of the reaction between chlorine dioxide and potassium iodide in aqueous solution. J. Am. Chem. Soc..

[77] Kern D.M., Kim C.-H. (1965). Iodine Catalysis in the Chlorite-Iodide Reaction1. J. Am. Chem. Soc..

[78] Lengyel I., Li J., Kustin K., Epstein I.R. (1996). Rate constants for reactions between iodine-and chlorine-containing species: A detailed mechanism of the chlorine dioxide/chlorite-iodide reaction. J. Am. Chem. Soc..

[79] Lee Y., Von Gunten U. (2012). Quantitative structure–activity relationships (QSARs) for the transformation of organic micropollutants during oxidative water treatment. Water Res..

[80] Ganiev I., Timergazin Q., Kabalnova N., Shereshovets V., Tolstikov G. (2005). Reactions of chlorine dioxide with organic compounds. Eur. Chem.-Technol. J..

[81] Amor H.B., De Laat J., Dore M. (1984). Mode d’action du bioxyde de chlore sur les composes organiques en milieu aqueux: Consommations de bioxyde de chlore et reactions sur les composes phenoliques. Water Res..

[82] Wajon J.E., Rosenblatt D.H., Burrows E.P. (1982). Oxidation of phenol and hydroquinone by chlorine dioxide. Environ. Sci. Technol..

[83] Andrés C.M.C., Pérez de la Lastra J.M., Juan C.A., Plou F.J., Pérez-Lebeña E. (2022). Hypochlorous Acid Chemistry in Mammalian Cells—Influence on Infection and Role in Various Pathologies. Int. J. Mol. Sci..

[84] Lin X., Zhang J., Luo X., Zhang C., Zhou Y. (2011). Removal of aniline using lignin grafted acrylic acid from aqueous solution. Chem. Eng. J..

[85] Guo Y., Xu J., Bai X., Lin Y., Zhou W., Li J. (2022). Free chlorine formation in the process of the chlorine dioxide oxidation of aliphatic amines. Water Res..

[86] Hull L., Davis G., Rosenblatt D., Williams H., Weglein R. (1967). Oxidations of amines. III. Duality of mechanism in the reaction of amines with chlorine dioxide. J. Am. Chem. Soc..

[87] Rosenblatt D., Hull L., De Luca D., Davis G., Weglein R., Williams H. (1967). Oxidations of amines. II. Substituent effects in chlorine dioxide oxidations. J. Am. Chem. Soc..

[88] Andrzejewski P., Nawrocki J. (2007). N-nitrosodimethylamine formation during treatment with strong oxidants of dimethylamine containing water. Water Sci. Technol..

[89] Rosenblatt D.H., Hayes A.J., Harrison B.L., Streaty R.A., Moore K.A. (1963). The reaction of chlorine dioxide with triethylamine in aqueous solution1. J. Org. Chem..

[90] Selbes M., Kim D., Karanfil T. (2014). The effect of pre-oxidation on NDMA formation and the influence of pH. Water Res..

[91] Lee C., Schmidt C.K., Yoon J., von Gunten U. (2007). Oxidation of N-nitrosodimethylamine (NDMA) precursors with ozone and chlorine dioxide: Kinetics and effect on NDMA formation potential. Environ. Sci. Technol..

[92] Yakupov M.Z., Shereshovets V.V., Imashev U.B., Ismagilov F.R. (2001). Liquid-phase oxidation of thiols with chlorine dioxide. Russ. Chem. Bull..

[93] Tan H., Wheeler W.B., Wei C.I. (1987). Reaction of chlorine dioxide with amino acids and peptides: Kinetics and mutagenicity studies. Mutat. Res..

[94] Ison A., Odeh I.N., Margerum D.W. (2006). Kinetics and mechanisms of chlorine dioxide and chlorite oxidations of cysteine and glutathione. Inorg. Chem..

[95] Darkwa J., Olojo R.O., Chikwana E., Simoyi R.H. (2004). Antioxidant Chemistry: Oxidation of l-Cysteine and Its Metabolites by Chlorite and Chlorine Dioxide. J. Phys. Chem. A.

[96] Napolitano M.J., Green B.J., Nicoson J.S., Margerum D.W. (2005). Chlorine dioxide oxidations of tyrosine, N-acetyltyrosine, and dopa. Chem. Res. Toxicol..

[97] Stewart D.J., Napolitano M.J., Bakhmutova-Albert E.V., Margerum D.W. (2008). Kinetics and mechanisms of chlorine dioxide oxidation of tryptophan. Inorg. Chem..

[98] Ogata N. (2007). Denaturation of protein by chlorine dioxide: Oxidative modification of tryptophan and tyrosine residues. Biochemistry.

[99] Noss C.I., Hauchman F.S., Olivieri V.P. (1986). Chlorine dioxide reactivity with proteins. Water Res..

[100] Rougé V. (2018). Chlorine Dioxide Oxidation in Water Treatment: Impact on Natural Organic Matter Characteristics, Disinfection Byproducts and Comparison with Other Oxidants.

[101] Bakhmutova-Albert E.V., Margerum D.W., Auer J.G., Applegate B.M. (2008). Chlorine dioxide oxidation of dihydronicotinamide adenine dinucleotide (NADH). Inorg. Chem..

[102] Bryan R.F. (1996). Biochemistry. Second Edition by D. Voet and J. G. Voet. Acta Crystallogr. Sect. D-Biol. Crystallogr..

[103] Rubio-Casillas A., Cambra-Madrid P. (2021). Farmacocinética y farmacodinamia del dióxido de cloro. E-CUCBA.

[104] Moore G.S., Calabrese E.J., DiNardi S., Tuthill R.W. (1978). Potential health effects of chlorine dioxide as a disinfectant in potable water supplies. Med. Hypotheses.

[105] Hagiwara Y., Inoue N. (2015). First case of methemoglobinemia caused by a ClO2-based household product. Pediatr. Int..

[106] Romanovsky A., Djogovic D., Chin D. (2012). A Case of Sodium Chlorite Toxicity Managed with Concurrent Renal Replacement Therapy and Red Cell Exchange. J. Med. Toxicol..

[107] Chejfec-Ciociano J.M., Martínez-Herrera J.P., Parra-Guerra A.D., Chejfec R., Barbosa-Camacho F.J., Ibarrola-Peña J.C., Cervantes-Guevara G., Cervantes-Cardona G.A., Fuentes-Orozco C., Cervantes-Pérez E. (2022). Misinformation About and Interest in Chlorine Dioxide During the COVID-19 Pandemic in Mexico Identified Using Google Trends Data: Infodemiology Study. JMIR Infodemiol..

[108] Mostajo-Radji M.A. (2021). Pseudoscience in the times of crisis: How and why chlorine dioxide consumption became popular in Latin America during the COVID-19 pandemic. Front. Political Sci..

[109] Burela A., Hernández-Vásquez A., Comandé D., Peralta V., Fiestas F. (2021). Dióxido de cloro y derivados del cloro para prevenir o tratar la COVID-19: Revisión sistemática. Rev. Peru. De Med. Exp. Y Salud Pública.

[110] Soriano-Moreno D.R., Fernandez-Guzman D., Ccami-Bernal F., Rojas-Miliano C., Nieto-Gutierrez W. (2021). Factors associated with the consumption of chlorine dioxide to prevent and treat COVID-19 in the Peruvian population: A cross-sectional study. BMC Public Health.

[111] (2020). 111. La AEMPS Advierte de los Riesgos Graves Para la Salud por el Consumo de Dióxido de Cloro o MMS. Agencia Española De Medicam. Y Prod. Sanit. (AEMPS). https://www.aemps.gob.es/informa/la-aemps-advierte-de-los-riesgos-graves-para-la-salud-por-el-consumo-de-dioxido-de-cloro-o-mms/.

[112] Vincenti S., de Waure C., Raponi M., Teleman A.A., Boninti F., Bruno S., Boccia S., Damiani G., Laurenti P. (2019). Environmental surveillance of Legionella spp. colonization in the water system of a large academic hospital: Analysis of the four-year results on the effectiveness of the chlorine dioxide disinfection method. Sci. Total Environ..

[113] Southwell K.L. (2003). Chlorine Dioxide Dry Fumigation in Special Collection Libraries. Libr. Arch. Secur..

[114] Buttner M.P., Cruz P., Stetzenbach L.D., Klima-Comba A.K., Stevens V.L., Cronin T. (2004). Determination of the Efficacy of Two Building Decontamination Strategies by Surface Sampling with Culture and Quantitative PCR Analysis. Appl. Environ. Microbiol..

[115] Hubbard H., Poppendieck D., Corsi R.L. (2009). Chlorine dioxide reactions with indoor materials during building disinfection: Surface uptake. Environ. Sci. Technol..

[116] Wilson S.C., Wu C., Andriychuk L.A., Martín J.M., Brasel T.L., Jumper C.A., Straus D.C. (2005). Effect of Chlorine Dioxide Gas on Fungi and Mycotoxins Associated with Sick Building Syndrome. Appl. Environ. Microbiol..

[117] Haynes W.M. (1990). CRC Handbook of Chemistry and Physics.

[118] Lowe J.J., Gibbs S.G., Iwen P.C., Smith P.W., Hewlett A.L. (2013). Impact of chlorine dioxide gas sterilization on nosocomial organism viability in a hospital room. Int. J. Environ. Res. Public Health.

[119] Burela A., Hernández-Vásquez A., Comandé D., Peralta V., Fiestas F. (2021). Chlorine dioxide and chlorine derivatives for the prevention or treatment of COVID-19: A systematic review. Rev. Peru. De Med. Exp. Y Salud Publica.

[120] Silva A.A., Orantes L.D.C., Enriquez K.O.G., Perez F.S.P., Basilio A., Jimenez Y.L.C., Díaz-Gutiérrez S.P. (2020). Chemical pneumonitis secondary to chlorine dioxide consumption in a patient with severe Covid-19. Platelets.

[121] Lardieri A., Cheng C., Jones S.C., McCulley L. (2021). Harmful effects of chlorine dioxide exposure. Clin. Toxicol..

